# Determination and risk assessment of aflatoxin B1 in the kernel of imported raw hazelnuts from Eastern Azerbaijan Province of Iran

**DOI:** 10.1038/s41598-024-57422-2

**Published:** 2024-03-22

**Authors:** Parnian Samimi, Ramin Aslani, Ebrahim Molaee-Aghaee, Parisa Sadighara, Nabi Shariatifar, Gholamreza Jahed Khaniki, Sibel Ozcakmak, Zahra Reshadat

**Affiliations:** 1 Division of Food Safety and Hygiene, Department of Environmental Health Engineering, School of Public Health, Tehran University of Medical Sciences, Tehran, Iran; 2 Provincial Directorate of Agriculture and Livestock, Ministry of Agriculture and Forestry, Samsun, Türkiye; 3 Department of Food Hygiene, Science and Research Branch, Islamic Azad University, Tehran, Iran

**Keywords:** Microbiology, Environmental sciences

## Abstract

Aflatoxin B1 (AFB1) is widespread and seriously threatens public health worldwide. This study aimed to investigate AFB1 in imported hazelnut samples in northwest of Iran (Eastern Azerbaijan Province) using High-Performance Liquid Chromatography with a Fluorescent Detector (HPLC-FLD). In all tested samples AFB1 was detected. The mean concentration of AFB1 was 4.20 μg/kg and ranged from 3.145 to 8.13 μg/kg. All samples contained AFB1 levels within the maximum acceptable limit except for one sample. Furthermore, the human health risk assessment of AFB1 from consuming imported hazelnuts by Iranian children and adults was evaluated based on the margin of exposure (MoE) and quantitative liver cancer risk approaches. The MoE mean for children was 2529.76, while for adults, it was 8854.16, indicating a public health concern. The present study found that the risk of developing liver cancer among Iranian children was 0.11100736 per 100,000 people, and in the Iranian adult population was 0.0314496 cancers per 100,000 people. Since environmental conditions potentially affect aflatoxin levels in nuts, countries are advised to monitor aflatoxin contents in imported nuts, especially from countries with a conducive climate for mold growth.

## Introduction

Hazelnut (*Corylus avellana*) is a hard-peeled fruit that grows predominantly in the Mediterranean basin (temperate climates with high humidity and rainfall) and is internationally traded. It is estimated that approximately 1,200,000 tons of hazelnuts are produced annually worldwide, and the majority are produced in Turkey, Italy, Azerbaijan, the United States, Spain, Greece, Georgia, England, and Iran^1,2^. Hazelnuts are a rich source of squalane, protein, carbohydrates, vitamins, minerals, and unsaturated fatty acids. It has been established that hazelnut oil reduces blood cholesterol levels and hypertension side effects. In addition, 100 g of hazelnuts provide 600–650 cal^3,4^. Since hazelnuts contain various nutrients, they are susceptible to decay and mold growth when harvested, dried, and stored at high temperatures and humidity^4,5^.

The prevalence of molds is one of the main challenges in the nut industry. Besides causing spoilage of crops, molds also produce mycotoxin. Mycotoxins are secondary metabolites of mold and are classified as food contaminants^6,7^. Aflatoxins (AFs) are the most prevalent type of mycotoxin in hazelnuts, peanuts, pistachio nuts, almonds, Brazil nuts, and walnuts^8,9^. These metabolites pose significant risks to human and animal health and agricultural output, leading to substantial economic losses^10,11^. AFs are among the most hazardous and toxic mycotoxins, primarily produced by *Aspergillus* species, notably *A. flavus* and *A. parasiticus*. Aspergillus fungi are capable of releasing diverse enzymes, which allows them to thrive under a variety of environmental conditions. AFs are stable metabolites and remarkably resistant to different processes^9,12,13^.

Since 1960, more than 20 AFs have been identified, and based on lethal dose values (LD_50_), aflatoxin B1 (AFB1) is the most toxic aflatoxin^14^. AFB1 is a genotoxic carcinogen linked to various diseases and complications, including acute hepatitis, liver cancer, immune and reproductive system disorders, and blood-forming stem cell dysfunction^3,15^.

Currently, numerous analytical techniques are available^16^, and previous researchers have utilized different methods to determine aflatoxin contents in foods, such as High-performance liquid chromatography (HPLC)^9,12^, Liquid Chromatography Coupled with Mass Spectrometry (LC–MS)^17^, Multidimensional Liquid Chromatography (2D-LC)^18^, Gas Chromatography (GC)^19^, Supercritical Fluid Chromatography (SFC)^20^, Enzyme-Linked Immunosorbent Assay–ELISA^21^, and UV–vis spectroscopic^22,23^.

Iran has an annual production of more than 50 thousand tons of hazelnuts and is among the top ten hazelnut producing countries. In recent years, the production of hazelnuts in Iran has decreased due to various reasons, including climate change, which has led to the annual import of a significant amount of this food item. Considering the high consumption of hazelnut kernels in Iran and the fact that no comprehensive study has been conducted on the aflatoxin concentration in imported hazelnuts in Iran. Therefore, the present study was carried out in order to determine the contents and assess the health risks of aflatoxin B1 in raw hazelnut kernels imported from Iran.

## Materials and methods

### Sample collection

In total, 60 samples (considering double sampling, each 500 g) of imported raw hazelnut kernels without shell were collected from Eastern Azerbaijan Province market, Iran during 2022. All samples were kept in the glass container with the condition of sterility in refrigerator (4 °C) until analyses at the same day.

### Chemical

All chemicals were of analytical grade and were purchased from Merck Co., (Darmstadt, Germany).

### Sample preparation and analysis

Immunoaffinity columns (IAC) were ordered from Neogen (Lansing, MI, USA). The AOAC Official Method 991.31 was employed to determine AFB1 hazelnut samples. Initially, 50 g of the hazelnut sample was ground with a blender, mixed with 5 g of NaCl from Merck (Darmstadt, Germany), and then placed into a suitable container. A digital scale with a sensitivity of 0.1 g was employed to weigh the samples. The sample containing NaCl was transferred to a mixing tank, and 50 mL of extracting solvent (MeOH from Merck (Darmstadt, Germany) and water in an 8:2 v/v ratio) was added to each sample and blended for 1 min. Then, the obtained solution was filtered via filter paper (Whatman No. 1). Afterward, 5 ml of the filtered solution was diluted with 35 ml of grade 3 water. The diluted solution was filtered again with microfiber filter paper, and 20 ml was applied to an HPLC immunoaffinity column. The immunoaffinity column was prepared utilizing antibodies of aflatoxins B and maintained at 25 °C until connecting to the tank. In the following step, 3 ml of PBS buffer (phosphate buffer saline) was dripped into the tank, and the solution flowed through the prepared immunoaffinity column at a rate of 2–3 ml per minute. The immunoaffinity column was washed with 20 ml (2 × 10 ml) of deionized water and dried with positive air pressure. The output mixture was collected in clean vials (HPLC-specific vials) after 375 L of methanol from Merck (Darmstadt, Germany) was passed through the column. The contents of the vials were diluted with 375 ml of deionized water, which was then stirred using a vortex (vacuum pump). The standard curve and calibration were prepared by injecting specific amounts of standard aflatoxins into the column before injection of the samples. The percent recovery of AFB1 was determined by injecting 200 μl of standard samples of AFB1 with a concentration of 5 ml into the HPLC (Alliance e2695, American Waters Company, fluorescence detector (FLR2475))^9,12,24^.

### Health risk assessment

Table 1 describes the details of the parameters used to compute the risk assessment caused by exposure to AFB1 from hazelnut consumption.

**Table 1:** Description of parameters used to calculate the risk assessment of AFB1 through the intake of hazelnut kernel.

Factor	Meaning	Unit	Values
C	Minimum concentration of AFB1	μg/kg	3.15
	Maximum concentration of AFB1	–	8.13
	Mean concentration of AFB1	–	4.20
IR	Ingestion rate	g/day	0.32
BW	Average body weight of adults	kg	70
	Average body weight of children	–	20
BMDL10	Benchmark dose lower confidence limit	ng/kg bw/day	170
HBsAg*	Prevalence positive of HBsAg^+^	–	2.2%
	Prevalence negative of HBsAg^-^	–	97.8%

*Hepatitis B surface antigen.

### Estimation of daily intake

The estimated daily intake (EDI) of AFB1 was obtained based on the mean, minimum, and maximum concentrations of AFB1 in hazelnut samples for Iranian children and adults. The following equation was employed to compute the estimated daily intake (EDI)^25–28^:

1 $$ {\text{EDI}} = {\text{C}} \times {\text{IR}} / {\text{BW}} $$

where C is the concentration of AFB1, IR is the ingestion rate (0.32 g/day), and BW is the average body weight (20 kg for children and 70 kg for adults).

### Risk characterization

Risk assessment was carried out with margin of exposure (MoE) approach recommended by EFSA^29^ and quantitative liver cancer risk approach recommended by FAO and WHO^30^.

MoE is the proportion of a toxicological reference point to a dose that leads to a low but measurable response. BMDL10 is the lowest dose that is 95% assured to cause no more than 10% cancer incidence in rodents. MoE values lower than 10,000 are considered a concern from a public health viewpoint. The following equations were used to calculate the margin of exposure (MoE)^31,32^:

2 $$ {\text{MoE}} = {\text{BMDL}} / {\text{EDI}} $$

The quantitative liver cancer risk approach was used to evaluate the liver cancer risk induced by exposure to AFB1. The probability of developing liver cancer resulting from exposure to AFB1 is synergistically augmented by hepatitis B. This risk assessment considers both the carcinogenic potency for individuals infected with hepatitis B and those not infected with hepatitis B. For hepatitis B surface antigen-positive individuals (HBsAg^+^) and hepatitis B surface antigen-negative individuals (HBsAg^−^), the carcinogenic potency of AFB1 is 0.3 and 0.01 cancers/year/10^5^ individuals per ng/kg bw/day. The prevalence of HBsAg^+^ in Iran is 2.2%^33^. The cancer risk of AFB1 was esteemed using the following Eqs.^31,34,35^:

3 $$ {\text{Pcancer}} = 0.01 \times \% {\text{HBsAg}} ^{ - } + 0.3 \times \% {\text{HBsAg}} + $$

4 $$ {\text{Cancer}} {\text{risk}} = {\text{EDI}} \times {\text{Pcancer}} $$

## Results

Hazelnuts were spiked with AFB1 to determine analytical accuracy and performance. The limit of detection (LOD) and limit of quantitation (LOQ) were 0.004 and 0.012 µg/kg, respectively. Also, the correlation coefficient (R^2^) of the dependent matrix calibration plots was 0.998%, and the Y-line equation was y = 3E + 07x-1848. The recovery rate of AFB1 was 113.1%.

**Figure 1:**
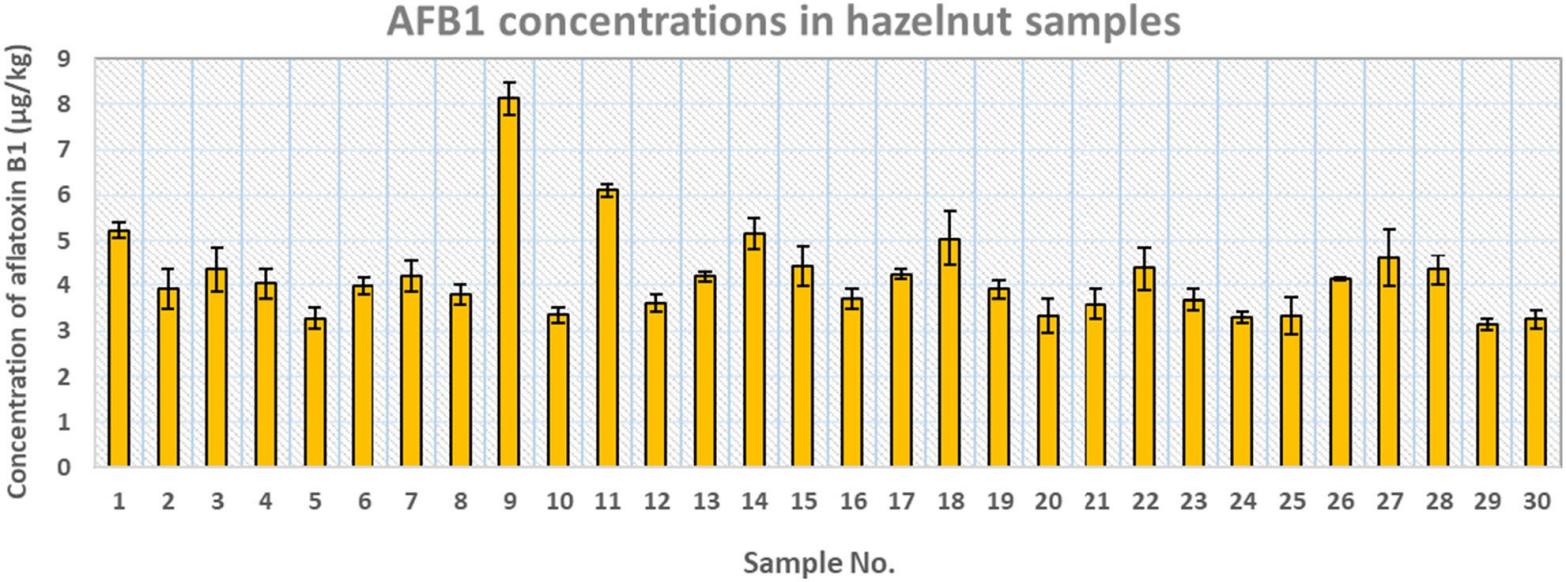
The concentration of aflatoxin B1 in 30 samples of hazelnut kernel.

Figure 1 illustrates the levels of aflatoxin B1 measured in 30 hazelnut samples collected from the Eastern Azerbaijan market, Iran. AFB1 was found in all samples at different amounts. The concentrations of AFB1 in the analyzed hazelnut samples ranged from 3.145 to 8.13 μg/kg, and the Mean ± SD concentration was 4.20 ± 0.15 μg/kg. AFB1 levels found in the analyzed samples were lower than the maximum permissible tolerance (8 μg/kg) recommended by Iran's National Standard (INSO No. 53925, Food and feed- Maximum tolerated level of mycotoxins), except in one sample (sample 9). The European Commission^36^ recommends a maximum acceptable level of AFB1 contamination in foods between 2 and 12 μg/kg, whereas the United States Food and Drug Administration (FDA) has established a limit of 20 μg/kg^9,37^.

## Discussion

As shown in Table 2, the AFB1 concentration found in the current investigation was compared with those reported in the previous literature. Saffari et al. reported AFB1 levels in 100 samples of hazelnuts within Iran's acceptable standard, and the concentration of AFB1 was significantly lower than in the current results^38^. In the study by Gürses, the mean concentration of AFB1 in the hazelnut sample was 34.4 µg/kg and ranged from 1 to 113 µg/kg, which was higher than the present results^39^. In Turkey, Bircan et al. analyzed 2784 samples of dried Figs. (2643), pistachios (28), hazelnuts (80), peanuts (10), and paprikas (23). They reported that 57% of pistachio, 2.5% of hazelnut, 50% of peanut,11.8% of dried figs, and 83% of paprika contained total aflatoxin in the range of 2.31–63.11 µg/kg, 5.46–6.55 µg/kg, 0.75–26.36 µg/kg, 0.2–162.76 µg/kg, and 0.38–14.71 µg/kg, respectively. Total AF levels in all pistachio and hazelnut samples were higher than the regulatory limits of the EU (5 µg/kg for AFB1 and 10 µg/kg for total AFs). Moreover, hazelnut samples contained less aflatoxin than the present study^40^. Kabak measured the aflatoxins concentration in 300 samples of hazelnuts and dried figs using the HPLC-FLD method. The study found that 6 raw hazelnut kernel samples and 5 roasted hazelnut kernel samples were positive for total aflatoxin. AFs concentration ranged from 0.09 to 11.3 µg/kg in raw hazelnut kernel and from 0.17 to 11.2 µg/kg in roasted hazelnut kernel. Also, AFs level in 3 hazelnut samples exceeded the European maximum limits^41^.

**Table 2:** Comparison of aflatoxin B1 in the hazelnut sample in the present study with literature.

Country	Sample	No	Concentration (µg/kg)		Method	References
			Mean	Range		
Iran	Hazelnut	30	4.20	3.145-8.13	HPLC-FLD	Present study
Turkey	Hazelnut	50	4.23	0.09–10.6	HPLC-FLD	Kabak^41^
China	Hazelnut	20	2.10	–	UPLC-MS/MS	Wang et al^42^.
Iran	Hazelnut	100	0.93	0.06–1.98	HPLC-FLD	Saffari et al^38^.
Iran	Hazelnut	20	0.73	0.24–3.5	ELISA	Akbar et al^43^.
Italy	Hazelnut	14	–	0.4–56.1	HPLC-FLD	Diella et al^44^.
Iran	Pistachio	33	0.35	0.30–0.40	HPLC-FLD	Bagheri et al^9^.
Iran	Walnuts	450	–	0.80–14.50	HPLC-FLD	Taghizadeh et al^45^.
Pakistan	Peanuts	20	2.37	0.32–28.98	HPLC-FLD	Asghar et al^46^.

The hazelnut hard shell is a good obstacle against fungal contamination. However, climatic and storage conditions can cause aflatoxin formation^47^. Acute exposure to aflatoxin may trigger abdominal pain, nausea, vomiting, and seizures, while chronic exposure can lead to hepatotoxicity, teratogenicity, and immunotoxicity. In developing countries, aflatoxin is one of the leading causes of hepatocellular carcinoma^48^.

Hazelnuts produced in different countries may contain varying amounts of AFs because the growth of *Aspergillus* species and the production of aflatoxin predominantly depend on environmental conditions. As reported in the study by Prelle et al. 66.7% of Turkish hazelnuts (mean contamination 0.33 mg/kg) and 35.9% of Italian hazelnuts (mean contamination 0.14 mg/kg) were contaminated with aflatoxin^17^. Furthermore, Ebrahimi et al. after conducting a global systematic review of aflatoxins contents in various nut samples, concluded that the average amounts of AFB1 in hazelnut samples from different countries were as follows: Italy (28.15 µg/kg) > Turkey (2.97 µg/kg) > Spain (0.51 µg/kg)^49^.

AF levels in hazelnuts can be affected by growing, harvesting, drying, and storing conditions^5^. Ozay et al. in a three-year study on factors influencing fungal and aflatoxin levels in Turkish hazelnuts during growth, harvest, drying, and storage, concluded that manual harvesting of ripe hazelnuts or harvesting hazelnuts into cloth by shaking the trees, utilizing jute rather than of nylon sacks, and mechanical drying technique would mitigate aflatoxin content in hazelnuts^36^. In regions that produce nuts traditionally, nuts are commonly dried out under the sunlight, thus becoming more exposed to environmental factors. In these regions, mechanical drying techniques can effectively contribute to preventing mold growth and AF contamination.

It has been determined that AFs concentration increases with the long-term storage of nuts. Also, damaged nuts are more susceptible to aflatoxin contamination^50^. For instance, the study by Bensassi et al. indicated that after 2 years of pistachio nuts storage in an extremely dry and aerated place, AFs contamination obviously occurred. Also, after 4 years of storage, the mean AFB1 concentration ranged from 2.7 to 12.7 µg/kg, beyond the maximum allowable limit. Hence, nuts should not be stored for long periods of time^51^. Furthermore, Gürses et al. found that controlling humidity and reducing the storage period minimizes aflatoxin formation in hazelnuts^50^.

The use of UV radiation, infrared ray roasting, cold plasmas, hot air, and citric acid are some of the most effective methods for mitigating AFs levels^3,52,53^. Furthermore, manual and mechanical separating, roasting, and membrane peeling can diminish AFs levels in hazelnuts. It seems that the majority of aflatoxin is concentrated in the membrane of the hazelnut (especially the inner membrane), and when membranes are peeled, 98% of the aflatoxin contaminant is removed^54^. The Özer study indicated AFB1 concentration in raw hazelnuts was 11.28 µg/kg, and after roasting, it decreased to 11.11 µg/kg. This amount was reduced to 0.23 µg/kg after peeling the membrane^55^. In contrast, Amiri et al. found no difference between raw and roasted hazelnuts regarding AFB1 amounts. In other words, if there is AF contamination, it is not reasonable to expect the unacceptable and higher levels of AFs to be reduced by processing and become acceptable. Quality assurance systems for nut industries are crucial to minimizing the growth of toxigenic mold and, thereby, the occurrence of mycotoxins, especially supplier qualifications^56^.

### Health risk assessment

A risk assessment was conducted to evaluate the health effects of AFB1. Estimated daily intake (EDI) and margin of exposure (MoE) through hazelnut consumption by children and adults are presented in Table 3. The EDI means of AFB1 was 0.0504 ng/kg bw/day (range 0.0672–0.13008 ng/kg bw/day) for children and 0.0192 ng/kg bw/day (range 0.0144–0.0371 ng/kg bw/day) for adults.

**Table 3:** EDI and MoE through hazelnut consumption by children and adults.

Risk Scenario	Children			Adults		
	Minimum	Maximum	Mean	Minimum	Maximum	Mean
EDI	0.0504	0.13008	0.0672	0.0144	0.0371	0.0192
MoE	3373.01	1306.88	2529.76	11,805.55	4582.21	8854.16

The MoE mean was 2529.76 for children and 8854.16 for adults. These results demonstrate a public health concern for Iranian children and adults associated with AFB1 exposure through imported hazelnut consumption. Similarly to the results of this study, the MoE values reported by Taghizadeh et al^45^. Blanco-Lizarazo et al^57^. and Leong et al^58^. were less than 10,000, which provides evidence of a public health concern. In contrast, MoE values reported by Renwick et al. were not more than 10,000^59^.

**Figure 2:**
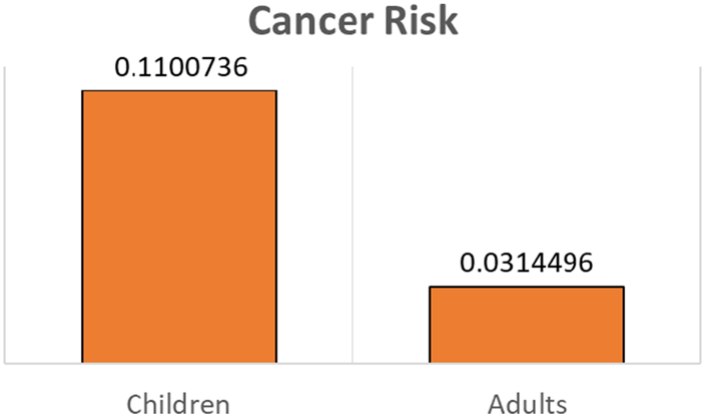
Liver cancer risk (cancers/year/ per 10^5^ individuals) through hazelnut consumption by Iranian children and adults.

Figure 2 illustrates the quantitative liver cancer risk incidence caused by AFB1 exposure based on imported hazelnut consumption. The risk of liver cancer in the Iranian children population was estimated at 0.1100736 cancers/year/ per 10^5^ individuals, and in the Iranian adult, it was 0.0314496 cancers/year/ per 10^5^ individuals. Results indicate that children have a significantly higher risk of liver cancer than adults.

Andrade et al. reported cancer risk for consumers with a high consumption level, and the entire population was 0.3056 and 0.0753 cancers/year/ per 10^5^ individuals, respectively^60^. Another investigation by Taghizadeh et al. revealed that risk of cancer in individuals who are positive for hepatitis B and those who are not positive for hepatitis B were 0.0001 and 0.0000034 cancers/year/ per 10^5^ individuals, respectively^45^. Undoubtedly, AFB1 can cause a severe public health threat, and the prevention and management of this contaminant should be a top priority.

The current study focused on a major contaminant AFB1 in a main nut with a relatively high consumption in food industry and also by people followed by a risk assessment. However, due to limitations in comparison with some studies, a combined samples of imported and domestic cultivated hazelnuts and collected from different provinces could reveal a more comprehensive state for AFB1 condition.

## Conclusion

The present investigation assessed AFB1 in imported hazelnuts into Iran. Except for one sample, all samples contained lower levels of AFB1 than the maximum permissible limit. The results obtained from margin of exposure risk assessment indicated a public health concern (MOE < 10,000 value). Due to the inherent properties of aflatoxins regarding their resistance to high temperatures, their complete removal from food matrices has faced significant challenges. It is recommended that aflatoxin levels in nuts are monitored constantly and accurately by appropriate government agencies.

## Data Availability

All data generated or analysed during this study are included in this published article.
